# Lauryl Gallate Activity and *Streptococcus mutans*: Its Effects on Biofilm Formation, Acidogenicity and Gene Expression

**DOI:** 10.3390/molecules25163685

**Published:** 2020-08-13

**Authors:** Vika Gabe, Mouhammad Zeidan, Tomas Kacergius, Maksim Bratchikov, Mizied Falah, Anwar Rayan

**Affiliations:** 1 Department of Physiology, Biochemistry, Microbiology and Laboratory Medicine, Institute of Biomedical Sciences, Faculty of Medicine, Vilnius University, Vilnius 03101, Lithuania; vika.gabe@mf.vu.lt (V.G.); tomas.kacergius@mf.vu.lt (T.K.); maksim.bratchikov@mf.vu.lt (M.B.); 2 Molecular Genetics and Virology Laboratory, Al-Qasemi Center of Excellence, Al-Qasemi Academic College, P.O. Box 124, Baka EL-Garbiah 30100, Israel; zeidanm@qsm.ac.il; 3 Institute for Medical Research, Galilee Medical Center, Nahariya 2210001, Israel; MiziedF@gmc.gov.il; 4 Faculty of Medicine in the Galilee, Bar-Ilan University, Safed 1311502, Israel; 5 The Institute of Applied Research-Galilee Society, P.O. Box 437, Shefa-Amr 20200, Israel; 6 Faculty of Science, Al-Qasemi Academic College, P.O. Box 124, Baka EL-Garbiah 30100, Israel

**Keywords:** lauryl gallate, dodecyl gallate, *Streptococcus mutans*, biofilm, acidogenicity, gene expression

## Abstract

*Streptococcus mutans* bacterium is implicated in the pathogenesis of dental caries due to the production of biofilm and organic acids from dietary sucrose. Despite the availability of various means of prophylaxis, caries still has a high worldwide prevalence. Therefore, it is important to find new pharmaceuticals to inhibit *S. mutans* biofilm formation and acidogenicity. The aim of the current study was to evaluate the activity of lauryl gallate (dodecyl gallate) against *S. mutans* acidogenicity, the expression of biofilm-associated genes, and biofilm development on solid surfaces (polystyrene, glass). The biofilm quantities produced by *S. mutans* bacteria were assessed using colorimetric and optical profilometry techniques. Acidogenicity was evaluated by measuring the pH of the biofilm growth medium with microelectrode. Assessment of the expression of gene coding for glucan-binding protein B (*gbpB*), glucosyltranferases B, -C, -D (*gtfB, -C, -D*), and the F-ATPase β subunit of F_1_ protein (*atpD*) was carried out using a quantitative reverse transcription-polymerase chain reaction (RT-qPCR). The results demonstrate the capacity of lauryl gallate to significantly inhibit *S. mutans* acidogenicity and biofilm development on solid surfaces, in a dose-dependent manner, compared to untreated bacteria (*p* < 0.05). The highest activity of lauryl gallate occurred at a concentration of 98.98 µM, at which it suppressed biofilm formation by 100% and lowered pH levels by 98%. The effect of lauryl gallate treatment on gene expression changes, as demonstrated by our RT-qPCR data, was limited to the *gtfD* gene only, was a significant (48%) decrease in gene expression, obtained for the biofilm-producing bacteria, while a 300% increase in fold change for the same gene occurred in the planktonic cells. It is important to note that in previous studies we showed a broader effect of related derivatives. However, a similar magnitude of difference in effects between biofilm and planktonic cells for the *atpD* gene was obtained after treatment with octyl gallate and reverse magnitude for the same gene after treatment with ethyl gallate. Therefore, to ascertain the possible direct or indirect effects of lauryl gallate, as well as octyl gallate and ethyl gallate, more research is needed to examine the effects on the amount of enzymes and on the enzymatic activity of the products of the affected genes that are involved in the production and maintenance of biofilm by *S. mutans.*

## 1. Introduction

*Streptococcus mutans* is an important bacterial species of human oral microbiota that predominantly colonizes the hard tissue surfaces of teeth, causing dental caries through sucrose-dependent adhesion and consequent biofilm formation [1,2]. This mechanism involves the utilization of dietary sucrose by the *S. mutans* glucosyltransferases, GtfB, -C, and -D, to produce water-insoluble, partly soluble, and soluble glucan polymers, respectively [3,4]. Interactions between the synthesized polymers and the *S. mutans* glucan-binding proteins GbpA, -B, -C, and -D, lead to the accumulation and development of dental biofilm [5]. Moreover, sucrose is further fermented by *S. mutans* bacteria to produce lactic acid, thereby lowering the pH to values below 5.0 within the biofilm [6,7]. This results in rapid acidification of the dental biofilm, which is subsequently sustained by the proton-translocating F-type ATPase of *S. mutans* [7,8]. This multimeric enzyme consists of the membrane-inserted F_0_ protein and peripherally attached F_1_ protein complexes, possessing the main functionally active b and β subunits encoded by *atpF* and *atpD* genes, respectively [8]. The activity of F-type ATPase maintains steady acidogenicity in the biofilm, which in turn causes the dissolution of tooth enamel with the development of carious lesions (i.e., dental caries) [6,9].

Like other bacteria that adopt this strategy, the biofilm of *S. mutans* confers resistance to pharmaceutical molecules not through gene transfer or specific mutations, as in classical antibiotic resistance, but due to its structural and functional repertoire. Biofilm aggregates consist of an extracellular polymeric matrix of complex and variable composition that contains exopolysaccharides, extracellular DNA, and other secreted proteins that can form amyloid fibers [10]. This macromolecular assemblage collectively blocks access by antimicrobial molecules and the components of host immune systems to biofilm-encased bacteria, which, thus, are inaccessible and resistant to treatment [11,12,13,14]. Besides protecting against antimicrobial compounds, the biofilm encapsulates bacteria and maintains homeostasis by retaining water to preserve a state of steady internal physical and chemical conditions that enable the bacteria to survive [10,13]. As for *S. mutans*, biofilms thus play a crucial role in chronic infections such as dental plaque and caries, and seem to play a role in the oral colonization associated with the severity of periodontal disease [15]. Therefore, it is important to develop new pharmaceutical molecules that can act against this critical formation of cariogenic biofilm, as dental caries are still prevalent in all age groups worldwide [16].

In recent years, due to the natural and unavoidable phenomenon of biofilm resistance, the search for new antibacterial molecules has been ongoing, because it is of especial importance to human health [11,17,18]. Active phytochemicals derived from natural sources have recently been the focus of the search for new molecules that act against bacterial pathogens [19,20,21,22,23,24,25]. Naturally occurring compounds in plants, as well as plant extracts, have been shown to exhibit anti-biofilm activity [24]. Among these natural compounds, epigallocatechin-3-gallate (EGCG), a major polyphenol in green tea (*Camellia sinensis*), also found in many other plants, has shown inhibitory effects against *Streptococcus mutans* [26]. In our previous studies, other natural derivatives of alkyl gallates that have been shown to significantly suppress *S. mutans* biofilm formation are methyl gallate (C1-MG) and ethyl gallate (C2-EG) [27,28], and we have demonstrated that an octyl gallate derivative (C8-OG) significantly diminished biofilm formation and suppressed the associated genes expressed in *S. mutans* [29]. Alkyl gallates with longer alkyl chains, e.g., the derivative lauryl gallate (dodecyl gallate), have yet to be investigated for these actions on biofilm formation and its related genes in *S. mutans*.

Lauryl gallate (C12-LG) is a natural plant triphenol, an N-alkyl ester derivative of gallic acid, that, like other derivatives (i.e., methyl, propyl, octyl gallates), is widely used as an antioxidant additive in food and in pharmaceutical and cosmetic products [30,31]. It protects cells against oxidative stresses caused by free radicals [30] and has been shown to have antiviral and antibacterial activity [32,33]. With its bulky polar head and single hydrophobic chain, lauryl gallate is likely to act on the superficial polar region and within the hydrophobic regions of bacterial membranes, modifying their fluidity, integrity, stability and bioactivity, and deactivating membrane proteins [34,35,36].

This study focused on the effects of lauryl gallate (C12-LG) against *S. mutans* biofilm formation on solid surfaces (polystyrene, glass) and on its acidogenicity and expression of biofilm-associated genes.

## 2. Results and Discussion

### 2.1. Antibacterial Activity of C12-LG on S. mutans

As shown in Table 1, the minimum inhibitory concentration (MIC) of C12-LG on *S. mutans* is 288.5 µM.

### 2.2. Inhibitory Effect of C12-LG Against the Formation of S. mutans Biofilm on Polystyrene Surfaces

**Figure 1:**
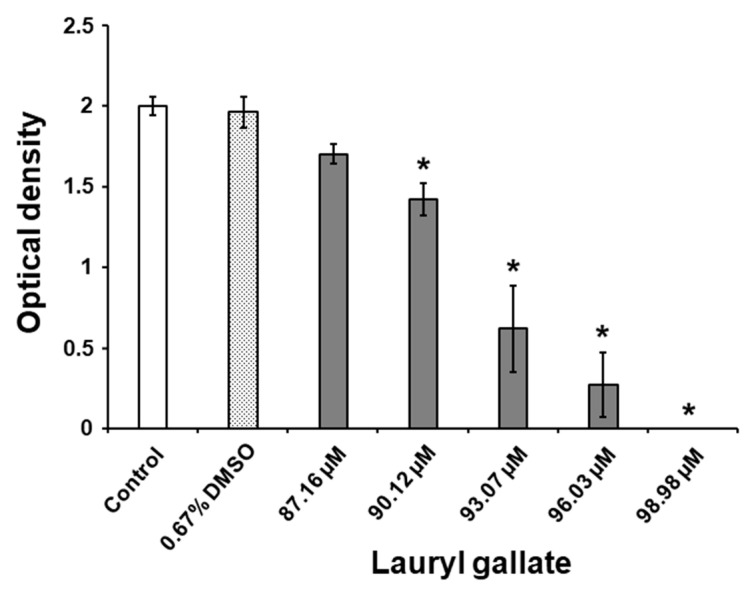
Activity of DMSO and the different C12-LG concentrations against the production of *S. mutans* biofilm biomass on polystyrene surfaces after 24 h of incubation in THB with 1% sucrose. Values are presented as the mean ± standard error obtained from three independent experiments (*n* = 3–9). The values denoted by an asterisk (*) are significantly different from those for the control group (*p* < 0.05).

An evaluation of biofilm biomass by colorimetric assay showed that C12-LG concentrations of 87.16, 90.12, 93.07, 96.03, and 98.98 µM inhibited *S. mutans* biofilm development on polystyrene surfaces in Todd Hewitt broth (THB) containing 1% sucrose (Figure 1). It is important to note that these biofilm-inhibiting concentrations fall below the identified MIC (i.e., 288.5 µM), indicating non-bactericidal activity for C12-LG. The suppressive effect of C12-LG against biofilm formation occurred in a dose-dependent manner. As shown in Figure 1, C12-LG concentrations from 90.12 to 98.98 µM significantly reduced *S. mutans* biofilm biomass on the polystyrene surfaces, in comparison to the biomass of the control bacteria (*p* < 0.05). It needs to be highlighted that the C12-LG concentration of 98.98 µM completely (i.e., by 100%) suppressed production of the biofilm biomass in comparison to that of the untreated bacteria. Moreover, DMSO alone, used at a concentration of 0.67%, had an insignificant effect against *S. mutans* biofilm development relative to that of the control bacteria (*p* > 0.05). Interestingly, the biofilm-inhibiting concentrations of C12-LG were in a quite narrow range, reflecting a similar finding for octyl gallate, that suppressive concentrations fell into a range from 97.4 to 100.24 µM [29]. However, this inhibitory effect of C12-LG occurred at lower concentrations than those of octyl gallate, and it was markedly greater than the activity of ethyl and methyl gallates, for which previously determined suppressive concentrations were in the ranges of 2.78–3.53 and 2.99–5.43 mM, respectively [27,28]. The results clearly indicate that the degree of the antibiofilm effect is positively correlated with the length of the alkyl chain in gallic acid esters, including lauryl gallate. Furthermore, in support of our findings, this type of suppressive activity, observed here in lauryl gallate, has also been noted by Zhang et al. [37] against another biofilm-forming pathogen, *Streptococcus pneumoniae*.

### 2.3. Inhibitory Effect of C12-LG Against the Formation of S. mutans Biofilm on Glass Surfaces

**Figure 2:**
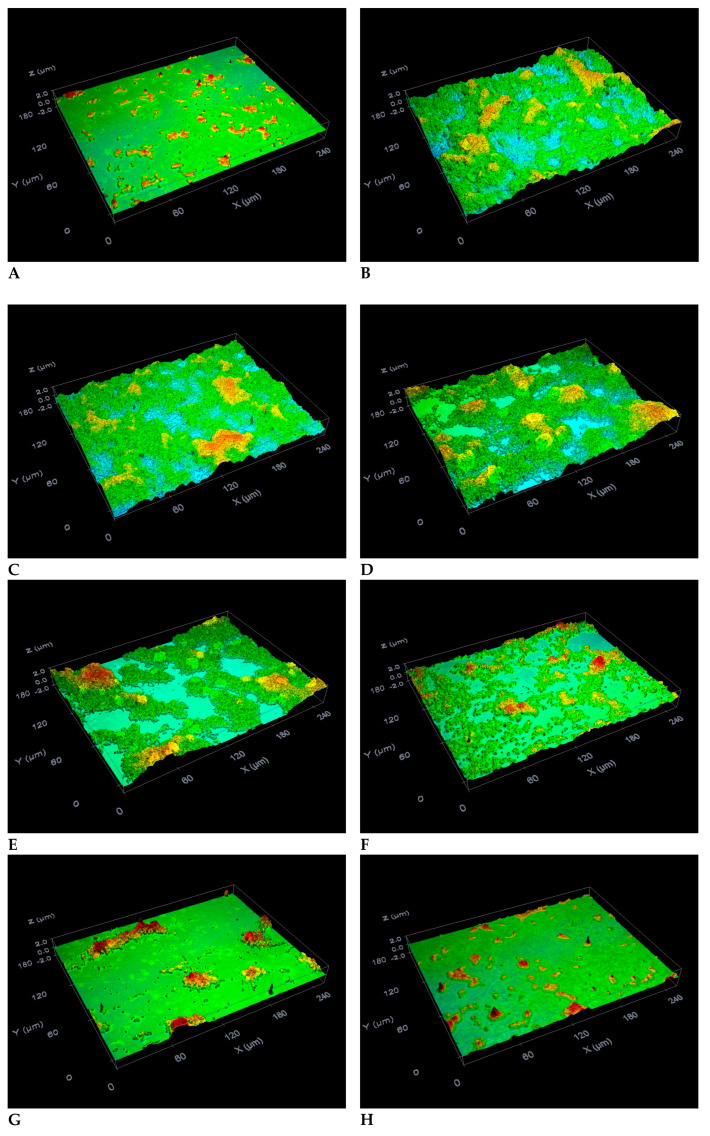
Activity of DMSO and C12-LG against *S. mutans* biofilm development on the glass surfaces after 24 h of incubation in THB. Shown are profilometric images of the glass slide surfaces covered by bacteria incubated (**A**) in the absence of C12-LG and sucrose, (**B**) without C12-LG, in the presence of 1% sucrose, and (**C**) treated with 0.67% DMSO or C12-LG at concentrations of (**D**) 87.16 µM, (**E**) 90.12 µM, (**F**) 93.07 µM, (**G**) 96.03 µM, and (**H**) 98.98 µM. Magnification: ×50.

**Figure 3:**
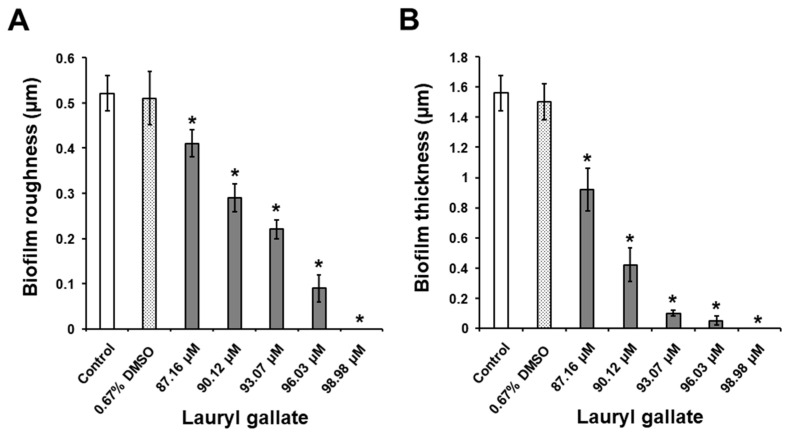
Activity of DMSO and the different C12-LG concentrations against biofilms produced by *S. mutans* on the glass surfaces after 24 h of incubation in THB with 1% sucrose. (**A**) The surface roughness parameter (*R*_q_) of the biofilm on the glass slides and (**B**) the biofilm thickness. Values are presented as the mean ± standard error obtained from three independent experiments (*n* = 18 for biofilm roughness; *n* = 15 for biofilm thickness). The values denoted by an asterisk (*) are significantly different from those for the control group (*p* < 0.05).

As seen in Figure 2B and Figure 3, the adhesion of *S. mutans* bacteria and consequent biofilm formation on the surfaces of the glass slide were strongly induced by the presence of 1% sucrose within the THB, as opposed to the adhesion of bacteria incubated in the absence of C12-LG and sucrose (Figure 2A). As regards the untreated bacteria, the values determined for the surface roughness (*R*_q_) and thickness parameters were 0.1 ± 0.01 and 0.15 ± 0.01 μm, respectively. A DMSO concentration of 0.67% insignificantly reduced biofilm formation in comparison to that of the control bacteria (*p* > 0.05) (Figure 2C and Figure 3). In contrast, exposing *S. mutans* bacteria grown in the presence of 1% sucrose to C12-LG concentrations of 87.16, 90.12, 93.07, 96.03, and 98.98 µM led to a dose-dependent inhibition of biofilm development on glass surfaces (Figure 2D–H, and Figure 3). In this regard, all of the C12-LG concentrations significantly decreased the surface roughness parameter (*R*_q_) of the biofilm (Figure 3A) and the biofilm thickness (Figure 3B) in comparison to those of the control bacteria (*p* < 0.05). Importantly, *S. mutans* biofilm formation on the glass surfaces was completely suppressed (100%) by a C12-LG concentration of 98.98 µM. These data are in agreement with results obtained in our recent studies [27,28,29], where it was found that other gallic acid esters (octyl, ethyl and methyl gallates) with shorter alkyl chain lengths also possess the capacity to inhibit *S. mutans* biofilm production on glass surfaces. However, in the present investigation, by using lower concentrations of lauryl gallate, we furnish strong evidence that longer alkyl chain lengths contribute to a stronger inhibitory activity of gallic acid esters against *S. mutans* biofilm formation on glass surfaces.

### 2.4. Inhibitory Effect of C12-LG Against the Acidogenicity of S. mutans Biofilm

The addition of 1% sucrose to THB and its subsequent breakdown by *S. mutans* bacteria caused a substantial acidogenicity of the biofilm grown in the absence of C12-LG, decreasing the pH levels of the biofilm growth medium by ~1.8-fold in the control group, compared to the pH levels in the blank group (Table 2). A DMSO concentration of 0.67% had no considerable effect on the acidogenicity of *S. mutans* biofilm, as shown in Table 2. In contrast, exposure of *S. mutans* bacteria to C12-LG concentrations ranging from 87.16 to 98.98 µM heightened the pH of the biofilm growth medium in a dose-dependent manner, approaching the pH levels of the blank group (Table 2). Moreover, it should be noted that all of the C12-LG concentrations significantly reduced the acidification of the biofilm in the treated *S. mutans* bacteria, relative to the untreated control bacteria (*p* < 0.05). The strongest effect of C12-LG occurred at a concentration of 98.98 µM, which lowered the pH level by 98%. Hence, lauryl gallate has the capacity to inhibit the production of organic acids (i.e., acidogenicity) in *S. mutans* biofilm. In contrast to the similar effects of octyl, ethyl and methyl gallates reported in our previous studies [27,28,29], lauryl gallate provided inhibitory activity against the acidogenicity of *S. mutans* biofilm at lower concentrations.

### 2.5. Gene Expression Analysis

**Figure 4:**
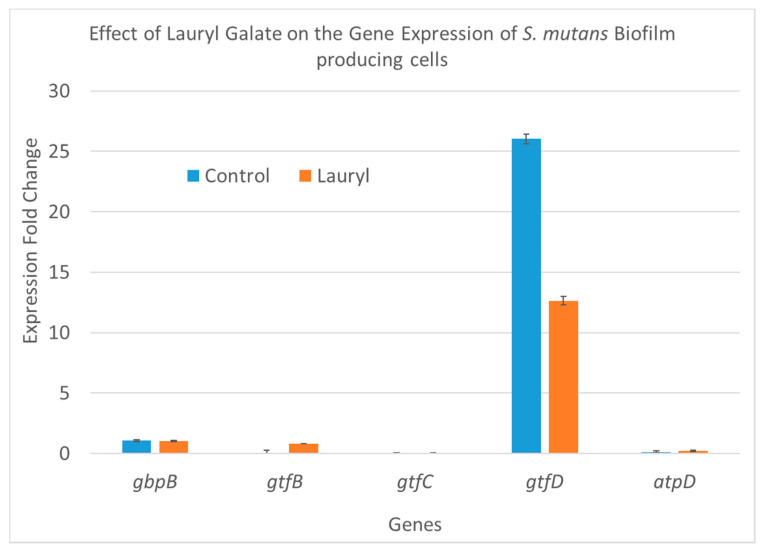
The effects of C12-LG treatment on the expression of five *S. mutans* genes involved in biofilm production. *S. mutans* cells were collected in the biofilm phase. C12-LG was applied at a 77.1 µM concentration (comparable to 1/4 of the MIC); its effect is shown in orange, while the bars in blue represent the values for the untreated biofilm-producing cells.

**Figure 5:**
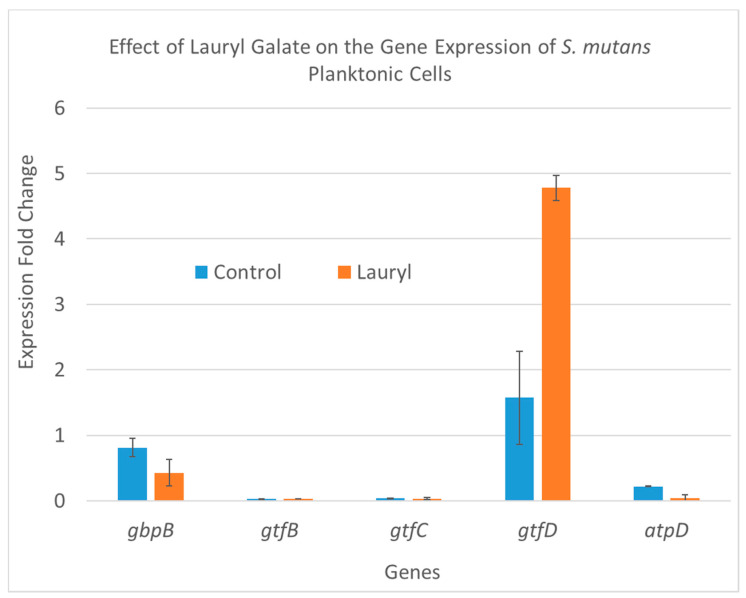
C12-LG effects on the expression of five *S. mutans* genes that are involved in biofilm production. The *S. mutans* cells were collected from planktonic growth cell populations. C12-LG was applied at a 77.1 µM concentration (comparable to 1/4 of the MIC). Its effects are shown in orange, while the bars in blue represent the values for the untreated controls.

To follow changes in gene expression, the two-step SYBR qRT-PCR was employed to determine relative changes in the expression of the *gbpB*, *gtfB*, *gtfC*, *gtfD*, and *atpD* genes involved in biofilm production, and *16SrRNA* was used as the reference gene. For this purpose, seeded *S. mutans* cells producing biofilm and planktonic cells were treated with three concentrations of C12-LG (288.5, 144.2, and 77.1 µM), and untreated cells were used as a control. Since the C12-LG 1.0 and 0.5 MIC samples showed no growth, or a negligible amount of *S. mutans*, only the 0.25 MIC sample was used for the gene expression analysis. The results of the analysis are shown in terms of gene expression fold changes in Table 3 and graphically presented in Figure 4 and Figure 5. In Figure 4, the biofilm-producing cells treated with C12-LG at a concentration of 77.1 µM (labeled “lauryl” and equal to 25% of the MIC value) show a significant (48%) decrease in fold change only for the *gtfD* gene and slight non-significant changes for the other four genes (*gbpB*, *gtfB*, *gtfC*, and *atpD*). In contrast, Figure 5 shows that C12-LG treatment of the planktonic cells caused a 300% increase in fold changes in the same gene, *gtfD,* which was down-regulated in the biofilm-producing cells. Concomitantly, in the other four genes, no remarkable change was noticed. Although in our previous studies [27,28,29], we reported on the broader effects of other gallic acid derivatives on the same five genes, it is interesting that in the current study, the C12-LG effect on gene expression in those genes was limited to the *gtfD* gene only. It is also important to note that the *gtfD* gene encodes to glucosyltransferase, which produces soluble glucan polymers. In the future, more studies will be conducted to study the effects of lauryl gallate on additional genes involved in biofilm production, as well as on the amount of proteins they produce.

## 3. Materials and Methods

### 3.1. The Source of Chemicals

Highly purified water was prepared with a Millipore Milli-Q Plus water purification system. Lauryl gallate, dimethyl sulfoxide (DMSO), and erythromycin were purchased from Sigma (Rehovot, Israel).

### 3.2. Test Bacterium and Culture Growth Conditions

The bacterium used in this study was *Streptococcus mutans* strain UA159 (ATCC No. 700610), obtained from the American Type Culture Collection (Manassas, VA, USA). This bacterial strain was cryo-preserved in 10% skim milk (Difco; BD BioSciences, Franklin Lakes, NJ, USA) at −70 °C until testing. Cultivation of *S. mutans* bacteria was performed in Bacto^™^ Todd Hewitt broth (THB; BD BioSciences, NJ, USA) under anaerobic conditions (95% N_2_ and 5% CO_2_) at 37 °C for 18 h prior to starting the experiments. Evaluation of the culture purity was carried out using Mitis Salivarius agar (Difco; BD BioSciences, NJ, USA) and Columbia agar, with 7% sheep blood (E&O Laboratories, Bonnybridge, Scotland).

### 3.3. Microdilution Test for Determining the Minimum Inhibitory Concentration (MIC)

To determine the MIC value, the broth microdilution assay was applied using a twofold serial dilution of C12-LG solution in brain heart infusion (BHI) broth, as described elsewhere [27,38]. Each well contained 10^5^ clone-forming units. The MIC value was defined as the lowest concentration that can inhibit the visible growth of bacteria in triplicate wells, following incubation at 37 °C for 24 h overnight. After the MIC value was visually determined, 20 µL of *p*-iodonitrotetrazolium violet (8 mg/mL in ethanol) were added to each well. The plate was incubated for another 30 min and inspected visually for any change in color from yellow to pink, which indicates a chemical reduction in dye due to bacterial growth. A twofold dilution of erythromycin was used as a positive control.

### 3.4. Experimental Procedures for S. mutans Biofilm Formation and Exposure to C12-LG

To determine the efficacy of C12-LG, *S. mutans* biofilm formation on polystyrene and glass surfaces was produced for the different experiments. Prior to starting the experiments, the optical density (OD) of the *S. mutans* culture was adapted to 0.2, which corresponded to 1.6 × 10^8^ bacterial cells/mL, using a microplate-reader spectrophotometer set to 630 nm. Twenty-four-well, flat-bottomed, polystyrene cell culture plates (Sarstedt, Nümbrecht, Germany), containing THB with 1% sucrose, were used to produce the biofilm on polystyrene surfaces. A solution of lauryl gallate (C12-LG; Sigma-Aldrich, Merck KGaA, Darmstadt, Germany) in pure dimethyl sulfoxide (DMSO; Sigma-Aldrich, Merck KGaA, Darmstadt, Germany) was added to the wells at final concentrations of 29.5 µg/mL (87.16 µM), 30.5 µg/mL (90.12 µM), 31.5 µg/mL (93.07 µM), 32.5 µg/mL (96.03 µM), and 33.5 µg/mL (98.98 µM). As the solvent for lauryl gallate, DMSO was added separately to the wells at the maximum concentration of DMSO that was used in the experiments, i.e., 0.67% (*v*/*v*). Sterile glass slides of 1-mm thickness, cut from standard microscope slides (76 × 26 mm; Thermo Fisher Scientific, Inc., Waltham, MA, USA) and inserted vertically into the plate wells, were used to provide the glass surfaces for the biofilm. Subsequently, *S. mutans* bacteria were inoculated into the wells at a final dilution of 1:100, and all of the plates were incubated anaerobically (95% N_2_ and 5% CO_2_) at 37 °C for 24 h. Afterwards, the biofilm produced on the polystyrene surfaces was assessed by using the colorimetric technique, whereas the biofilm developed on the glass surfaces was evaluated by applying the optical profilometry technique. In the experimental procedures, plate wells without *S. mutans* bacteria were used as blank controls, and unexposed *S. mutans* bacteria served as experimental controls. For the colorimetric and optical profilometry techniques, a percentage of C12-LG inhibition against biofilm formation was calculated using the values of OD, surface roughness (*R*_q_), and biofilm thickness parameters, according to the Equation (1):

(1) $$C12\text{-}\mathrm{LG}\;\mathrm{inhibitory}\;\%\;=\left(\frac{{\mathrm{Parameter}}_{unexposed\;control}-{\;\mathrm{Parameter}}_{exposure}}{{\mathrm{Parameter}}_{unexposed\;control}}\right)\times 100\%\;\;\;\;\;.$$

### 3.5. Colorimetric Technique

The biofilm biomass produced by *S. mutans* bacteria on polystyrene surfaces was quantified using the colorimetric technique, as reported in our previous studies [27,28]. In brief, following the staining of the fixed and air-dried biofilm with a 0.01% crystal violet solution (Merck KGaA, Darmstadt, Germany), the bound dye was released by applying a 33% acetic acid solution (Merck KGaA, Darmstadt, Germany). Then, the absorbance (i.e., OD) of the released dye solution from the samples was measured using a microplate-reader spectrophotometer set to 595 nm. Background staining was corrected for by subtracting the degree of staining in the blank wells and in the wells with unexposed bacteria grown in the absence of sucrose.

### 3.6. Optical Profilometry Technique

The quantities of biofilm formed by *S. mutans* bacteria on the glass surfaces were assessed by the optical profilometry technique, as described previously [27,28]. Briefly, the biofilm-covered and air-dried glass slides were evaluated applying a non-contact optical imaging profilometer Sensofar PLµ 2300 system (Terrassa, Spain), equipped with a 50× confocal objective spanning a view field of 253 × 190 μm. A total of six scanning measurements were carried out in order to evaluate the surface roughness, while five scanning measurements were conducted to assess the biofilm thickness per slide, halfway from the bottom to the top of the visible biofilm. Afterwards, the data for the scanned and measured samples were processed by using Gwyddion software (version 2.50, Department of Nanometrology, Czech Metrology Institute, Brno, Czech Republic; http://gwyddion.net) to compute the parameters for surface roughness and biofilm thickness. Calculation of the root mean square of the roughness (*R*_q_) parameter was performed in order to quantify the slide surface roughness, which signified the adhesion of *S. mutans* bacteria. The height of an artificially produced vertical scratch on each bacteria-covered slide was measured to quantify the biofilm thickness, which is an indicator of the level of maturity of the *S. mutans* biofilm. The background for the parameters of surface roughness and biofilm thickness was corrected for by subtracting the *R*_q_ and thickness values of the blank glass slides, and those of the glass slides with unexposed bacteria grown in the absence of sucrose.

### 3.7. Assessment of the Biofilm Acidogenicity

The acidogenicity of *S. mutans* biofilm was evaluated using the procedures outlined in our recent study [27]. Measurement of the pH of the biofilm growth medium was performed by using a microelectrode InLab^®^ Micro Pro ISM^®^ connected to a bench-top pH meter, a SevenCompact^™^ S210-Bio (Mettler-Toledo GmbH, Greifensee, Switzerland). The microelectrode was calibrated with standard pH buffers (pH 4.01 and 7.00).

### 3.8. Analysis of Gene Expression

Overnight cultures of *S. mutans* grown in Luria–Bertani (LB) broth were diluted into a fresh LB medium of 1% sucrose to obtain a final concentration of 0.5 × 10^5^ clone-forming units per milliliter (CFU/mL) and were equally distributed into 50-mL tubes (30 mL/tube). Lauryl gallate solution was added to the tubes at concentrations of 288.5, 144.2, and 77.1 µM. Cultures without lauryl gallate were used as a control. Cells were grown in three wells of a six-well plate at 5 mL per well (15 mL total), and the plates were incubated at 37 °C for 24 h. Planktonic cells were collected separately and stored at −4 °C for further analysis. The attached cells (biofilm) were scraped from the wells and stored at −4 °C for RNA isolation.

### 3.9. RNA Isolation

Total RNA was extracted in the manner previously reported [29]. In short, planktonic cells maintained in liquid culture before and after treatment with C12-LG were transferred separately to new 50 mL tubes. Then, biofilm-producing cells were collected by scraping the bottom of the culture tubes and transferred to separate 50 mL tubes. After both the planktonic and biofilm-produing cells were centrifuged at 4800× *g* for 10 min, the bacterial pellets were washed three times with 500 µL of ice-cold phosphate buffer solution (PBS). The RNA was extracted after subjecting the cell pellets to three cycles of re-suspension in CTAB/PVP/BME solution, followed by deep freezing at −80 °C and thawing at room temperature. The lysed cells were subsequently processed as described previously [27]; however, after DNAse treatment with a TURBO DNA-free kit, a phenol/chloroform/IAA step was included, followed by two cycles of chloroform/IAA extractions. RNA was collected on Qiagen spin columns and eluted in 50 microliters of RNase-free water following the Qiagen protocol. The quantity and purity of the total RNA samples were assessed by ultraviolet spectroscopy with a DS-11 spectrophotometer (DeNovix, Inc., Wilmington, North Carolina, USA).

### 3.10. RT-qPCR for the Estimation of Biofilm-Associated Gene Expression Following C12-LG Treatment

One microgram of total RNA was subjected to cDNA synthesis using the random hexamer primer included in the qPCRBIO cDNA Synthesis Kit (PCR Biosystems, Ltd., London, England). The qPCR was performed using a Biorad CT030008 system (Biorad,) with SSo SyGreen Mix (Biorad Ltd., London, England). One µL of each cDNA sample was used in 20 µL of total reaction volume. The final primer concentration was 400 nM for both the forward and reverse primers, in accordance with the manufacturer’s instructions. The cycling conditions were as follows: 30 s of initial denaturation at 95 °C, 40 cycles of 30 s at 95 °C and 30 s at 60 °C, and a final melting curve program. The primer sequences were as previously published [27].

Amplifications using total RNA that was not reverse-transcribed were performed to check for genomic DNA contamination, and no-template controls were included. The comparative ∆∆C_T_ Livak method for qPCR data was applied as a standard procedure in the analysis of the relative gene expression data. The *C*_T_ (cycle threshold) values were normalized to the reference gene 16S rRNA, and the difference in the ∆*C*_T_ values (∆∆*C*_T_) between the samples of interest and the control samples was calculated [29].

### 3.11. Statistical Analysis

The Statistical Package for the Social Sciences software, version 23.0 for Windows (IBM Corp., Armonk, NY, USA), was applied to analyze the data collected from the colorimetric, profilometric, and pH measurements. The intergroup differences (i.e., between the unexposed control group and the exposed groups) were estimated by using a one-way analysis of variance (ANOVA), followed by a post-hoc, least-significant difference test to compare the multiple means. The values are expressed as the mean ± standard error. A *p* value of less than 0.05 was considered statistically significant. To investigate gene expression, three independent experiments with two technical tests were conducted for each treatment (*n* = 6). The statistical analysis was performed using a one-way analysis of variance, followed by the Tukey–Kramer test, at a significance level of 0.05. The figures display the mean and standard deviations.

## 4. Conclusions

The accumulation of biofilm produced by *Streptococcus mutans* bacteria on hard tooth tissues remains one of the most prevalent oral diseases, so that the development of new antibiofilm agents is of critical importance. Our results show that exposure to C12-LG significantly diminished biofilm formation by *S. mutans* on solid surfaces and suppressed acidogenicity in a dose-dependent manner, compared to unexposed bacteria (*p* < 0.05). C12-LG concentration of 98.98 µM inhibited *S. mutans* biofilm development on solid surfaces by 100% and prevented any decrease in pH levels. The capability of C12-LG to inhibit biofilm formation and acidogenicity recommends it as an anticaries agent for oral formulations that aim to reduce the prevalence of dental caries. It is worth noting that the current available toxicological evidence indicates that gallic acid derivatives (ethyl gallate, propyl gallate, octyl gallate, and lauryl gallate) may be used safely as antioxidants. No toxic effects were observed at a dose level of 1000 mg/kg feed and toxic effects were found only at 3000 mg/kg feed or higher levels. The FAO/WHO Joint Expert Committee on Food Additives (JECFA) in 1976 “established an acceptable daily intake for man of 0.2 mg/kg body weight (as a sum of propyl, octyl and dodecyl gallates)” [39].

In the current study, we investigated the effects of C12-LG treatment on five important genes involved in biofilm production by *S. mutans*. The results show that biofilm-producing bacteria treated with C12-LG at a concentration of 77.1 µM show a significant (48%) decrease in fold change for the *gtfD* gene only, while no significant change was observed in the other four genes (*gbpB*, *gtfB*, *gtfC*, and *atpD*). However, in the planktonic cells, C12-LG treatment caused a 300% increase in fold change for the same gene, *gtfD,* which was down-regulated in the biofilm-producing cells. In the other four genes, no remarkable change was noted. Different expression changes of a similar magnitude between biofilm-producing and planktonic cells were shown in our previous studies [27,28,29] for the *atpD* gene after treatment with C8-OG. However, a reverse magnitude was shown for the same gene, *atpD,* after treatment with C2-EG. Nevertheless, our results show some expression change in genes involved in the production and maintenance of biofilm after treatment with gallic acid derivatives. However, to look for other possible effects of these derivatives, more studies are needed to ascertain the amount and enzymatic activities of the products of these genes.

## Figures and Tables

**Table 1:** Antibacterial activity of lauryl gallate (stock solution 100 mg/mL dissolved in pure dimethyl sulfoxide (DMSO)), erythromycin (positive control, stock solution 10 mg/mL dissolved in DMSO), and DMSO (solvent).

Compound	MIC
Lauryl gallate	288.5 µM
Erythromycin	6.54 µM
DMSO	25% (*v*/*v*)

**Table 2:** Effects of DMSO and the different C12-LG concentrations on the pH levels of the *S. mutans* biofilm growth medium after 24 h of incubation in the presence of 1% sucrose.

Experimental Group	pH
Blank	7.36 ± 0.03 *
Control	4.16 ± 0.01
DMSO (0.67%)	4.18 ± 0.03
C12-LG (87.16 µM)	4.55 ± 0.04 *
C12-LG (90.12 µM)	4.75 ± 0.11 *
C12-LG (93.07 µM)	5.94 ± 0.35 *
C12-LG (96.03 µM)	6.32 ± 0.3 *
C12-LG (98.98 µM)	7.23 ± 0.03 *

Values are presented as the mean ± standard error obtained from three independent experiments (*n* = 3–9). The values denoted by an asterisk (*) differ significantly from those of the control group (*p* < 0.05).

**Table 3:** C12-LG effects on the expression of five genes from planktonic *S. mutans* cells and biofilm *S. mutans* cells.

**Planktonic *S. mutans* Gene Expression**						
		*gbpB*	*gtfB*	*gtfC*	*gtfD*	*atpD*
Average Fold Change	Control	0.812	0.026	0.04	1.575	0.219
	Lauryl	0.429	0.028	0.034	4.78	0.041
SD fold change	Control	0.199	0.003	0.012	0.191	0.049
	Lauryl	0.139	0.002	0.005	0.712	0.006
**Biofilm *S. mutans* Gene Expression**						
		*gbpB*	*gtfB*	*gtfC*	*gtfD*	*atpD*
Average Fold Change	Control	1.06	0.037	0.022	26.026	0.127
	Lauryl	1.025	0.811	0.076	12.634	0.204
Standard Deviation	Control	0.054	0.015	0.001	0.356	0.048
	Lauryl	0.098	0.218	0.009	0.394	0.086
